# The surgical treatment of non-metastatic melanoma in a Clinical National Melanoma Registry Study Group (CNMR): a retrospective cohort quality improvement study to reduce the morbidity rates

**DOI:** 10.1186/s12885-020-07705-4

**Published:** 2021-01-05

**Authors:** Antonella Vecchiato, Simone Mocellin, Paolo Del Fiore, Giulio Tosti, Paolo A. Ascierto, Maria Teresa Corradin, Vincenzo De Giorgi, Giuseppe Giudice, Paola Queirolo, Caterina Ferreli, Marcella Occelli, Monica Giordano, Giusto Trevisan, Luigi Mascheroni, Alessandro Testori, Romina Spina, Alessandra Buja, Francesco Cavallin, Corrado Caracò, Antonio Sommariva, Carlo Riccardo Rossi, on behalf of the Clinical National Melanoma Registry Study Group at the Italian Melanoma Intergroup (IMI), Salvatore Asero, Rosanna Barbati, Luca Bianchi, Francesca Bruder, Caterina Catricalà, Saverio Cinieri, Michele Del Vecchio, Franco Di Filippo, Maria Concetta Fargnoli, Maria Teresa Fierro, Rosachiara Forcignanò, Massimo Guidoboni, Carmelo Iacono, Lucia Lospalluti, Michele Maio, Laura Milesi, Gianmichele Moise, Giovanna Moretti, Riccardo Pellicano, Maria Antonietta Pizzichetta, Gaetana Rinaldi, Giovanni Sanna, Stefania Staibano, Marilena Visini, Guido Zannetti, Leonardo Zichichi

**Affiliations:** 1 Surgical Oncology Unit, Veneto Institute of Oncology IOV – IRCCS, Padova, Italy; 2 Department of Surgery, Oncology and Gastroenterology (DISCOG), University of Padua, Padova, Italy; 3 Division of Melanoma, Sarcoma and Rare Tumors, IRCCS, European Institute of Oncology, Milan, Italy; 4 Department of Melanoma and Cancer Immunotherapy, Istituto Nazionale Tumori IRCCS Fondazione Pascale, Naples, Italy; 5 Department of Dermatology, Santa Maria degli Angeli Hospital, Pordenone, Italy; 6 Section of Dermatology, Department of Health Sciences, University of Florence, Florence, Italy; 7 Division of Plastic and Reconstructive Surgery and Burn Unit, University of Bari, Bari, Italy; 8 Division of Medical Oncology for Melanoma, Sarcoma, and Rare Tumors, IEO, European Institute of Oncology IRCCS, Milan, Italy; 9 Section of Dermatology, Department of Medical Sciences and Public Health, University of Cagliari, Cagliari, Italy; 10 Medical Oncology Unit, Santa Croce and Carle Teaching Hospital, Cuneo, Italy; 11 Pathology, ASST-Lariana, Ospedale Sant’Anna, Como, Italy; 12 DSM-Department of Medical Sciences, University of Trieste, Trieste, Italy; 13 Unit of General Surgery, San Pio X Hospital, Milan, Italy; 14 Department of Dermatology, Fondazione IRCCS Policlinico San Matteo, 27100 Pavia, Italy; 15 Department of Cardiological, Thoracic and Vascular Sciences, and Public Health, University of Padova, Padova, Italy; 16 Independent Statistician, Solagna, Italy; 17 National Cancer Institute Fondazione G. Pascale, SC Chirurgia Melanoma e dei Tumori Cutanei, Naples, Italy; 18 Unit of Surgical Oncology of the Esophagus and Digestive Tract, Veneto Institute of Oncology IOV-IRCCS, Padua, Italy

**Keywords:** Melanoma, Melanoma morbidity, Skin Cancer, Melanoma morbidity, Melanoma surgical treatment, Melanoma quality improvement

## Abstract

**Background:**

Reproducible, high-quality surgery is a key point in the management of cancer patients. Quality indicators for surgical treatment of melanoma has been presented with benchmarks but data on morbidity are still limited. This study presents the quality indicators on morbidity after surgical treatment for non-metastatic skin melanoma in an Italian registry.

**Methods:**

Data were extracted from the Central National Melanoma Registry (CNMR) promoted by the Italian Melanoma Intergroup (IMI). All surgical procedures (WE, SNLB or LFND) for non-metastatic skin melanoma between January 2011 and February 2017 were evaluated for inclusion in the study. Only centers with adequate completeness of information (> 80%) were included in the study. Short-term complications (wound infection, dehiscence, skin graft failure and seroma) were investigated.

**Results:**

Wound infection rate was 1.1% (0.4 to 2.7%) in WE, 1.3% (0.7 to 2.5%) in SLNB and 4.1% (2.1 to 8.0%) in LFND. Wound dehiscence rate was 2.0% (0.8 to 5.1%) in WE, 0.9% (0.2 to 3.0%) in SLNB and 2.8% (0.9 to 8.6%) in LFND. Seroma rate was 4.2% (1.5 to 11.1%) in SLNB and 15.1% (4.6 to 39.9%) in LFND. Unreliable information was found on skin graft failure.

**Conclusions:**

Our findings contribute to available literature in setting up the recommended standards for melanoma centers, thus improving the quality of surgery offered to patients. A consensus on the core issues around surgical morbidity is needed to provide practical guidance on morbidity prevention and management.

**Supplementary Information:**

The online version contains supplementary material available at 10.1186/s12885-020-07705-4.

## Background

Worldwide, 287,723 new cases of cutaneous melanoma are diagnosed each year, causing the death of 60,712 patients [1]. In non-metastatic melanoma (i.e. without clinically evident regional lymph node or distant metastasis), surgery is the mainstay of treatment [2]. The surgical strategy involves a combination of wide excision (WE), sentinel lymph node biopsy (SLNB) and radical lymph node dissection (LFND), according to cancer staging. WE can be virtually part of the surgical management of all patients with skin melanoma, SNLB is recommended based on the primary tumor thickness and LFND may be required in patients with positive sentinel node [3].

Reproducible, high-quality surgery is a key point in the management and prognosis of cancer patients [4, 5]. When a standardized surgical procedure is established, a cyclic audit (including collection, analysis and feedback of both procedural and outcome data) allows for monitoring and improving the quality of surgery. However, cancer surgery is not always standardized, thus limiting the application of such approach [6].

While quality of surgery has been already investigated in other cancers, the use of measures of quality assurance for surgery is less established in melanoma [6]. Available literature offers substantial heterogeneity in surgical procedures among melanoma centers or even among surgeons within the same center [7]. Although adherence to current standards is part of a quality assurance process, the spreading of clinical practice guidelines is not sufficient per se to warrant homogeneity and quality of surgical treatment [8]. There is a growing interest in the implementation of a quality assurance program that includes a quantitative analysis of a set of quality indicators, such as those that can be extracted from electronic medical records [6].

Since 2014, the Italian Melanoma Intergroup (IMI) has been promoting the standardization and quality control of surgical treatment of stage I-III melanoma in Italy [9]. The IMI achieved an expert consensus on surgical treatment of melanoma, defining a list of quality indicators (detection rate, false negative rate, minimum number of excised lymph nodes, postoperative morbidity and local recurrence) with reference to benchmark values, which could be the basis for a standardized quality assurance program in Italy [10]. While quality indicators for detection rate, false negative rate and minimum number of excised lymph nodes were presented for the Central National Melanoma Registry (CNMR), data on morbidity were still pending and data on recurrence are waiting for adequate follow-up [10].

The present study focuses on evaluation of the quality indicators about morbidity after surgical treatment for non-metastatic skin melanoma.

## Methods

This is a prospective multicenter study on postoperative morbidity after surgical treatment for non-metastatic skin melanoma. Data were extracted from the Central National Melanoma Registry (CNMR), which is a prospectively maintained national database for melanoma treatment promoted by the IMI in 43 melanoma centers in Italy [9]. The study was conducted according to Helsinki Declaration principles and was approved by the Ethics Committee of the Central National Melanoma Registry (CNMR). All patients gave their consent to have their data collected for scientific purpose.

The following short-term (within 30 days after surgery) complications were investigated: infection, dehiscence, skin necrosis after WE; infection, dehiscence and seroma after SLNB or LFND. Complications were defined using the National Cancer Institute Common Terminology Criteria for Adverse Events v4.0 [11]. Wound infection was defined as a disorder characterized by an infectious process involving the wound. Wound dehiscence was defined as a finding of separation of the approximated margins of a surgical wound. Skin necrosis was defined as a death of cells in living tissue caused by external factors such as infection, trauma, or bacterial agents. Seroma was defined as finding of a collection of serum in the tissues. Patient demographics were also collected.

All surgical procedures (WE, SNLB or LFND) for non-metastatic skin melanoma between January 2011 and February 2017 were evaluated for inclusion in the study. Only centers with adequate completeness of information (> 80%) were included in the study (11 centers for analysis of morbidity after WE; 14 centers for analysis of morbidity after SNLB; 11 centers for analysis of morbidity after LFND). Within selected centers, patients with pending information on morbidity and those with unfilled form were excluded from the analysis. Patient selection is shown in Supplementary Figs. 1, 2 and 3.

Statistical analysis was conducted using R 3.5 (R Foundation for Statistical Computing, Vienna, Austria) [12]. Categorical data were expressed as number and percentage, and continuous data as median and interquartile range (IQR). The morbidity rate was pooled among centers using a generalized linear mixed-effects model (with the center as random effect). The I^2^ value was used to investigate heterogeneity, and meta-regression was used to explore the role of center size and groin site on heterogeneity. A *p*-value less than 0.05 was considered statistically significant.

## Results

### Wide excision

The analysis included 3118 patients (1647 males and 1471 females) who underwent WE in 11 centers (median 246 patients/center; min 90 patients/center, max 650 patients/center). Median age was 57 years (IQR 45–70) and median BMI was 24.8 kg/m^2^ (IQR 22.4–27.5). Comorbidities were present in 459 patients (15.1%), while the information was not available in 80 patients. Overall, median Charlson Comorbidity Index was 2 (IQR 0–3).

Wound infection occurred in 44 patients and dehiscence in 100. The information on skin graft failure was not reliable due to the non-negligible number of unfilled forms (21%).

**Fig. 1:**
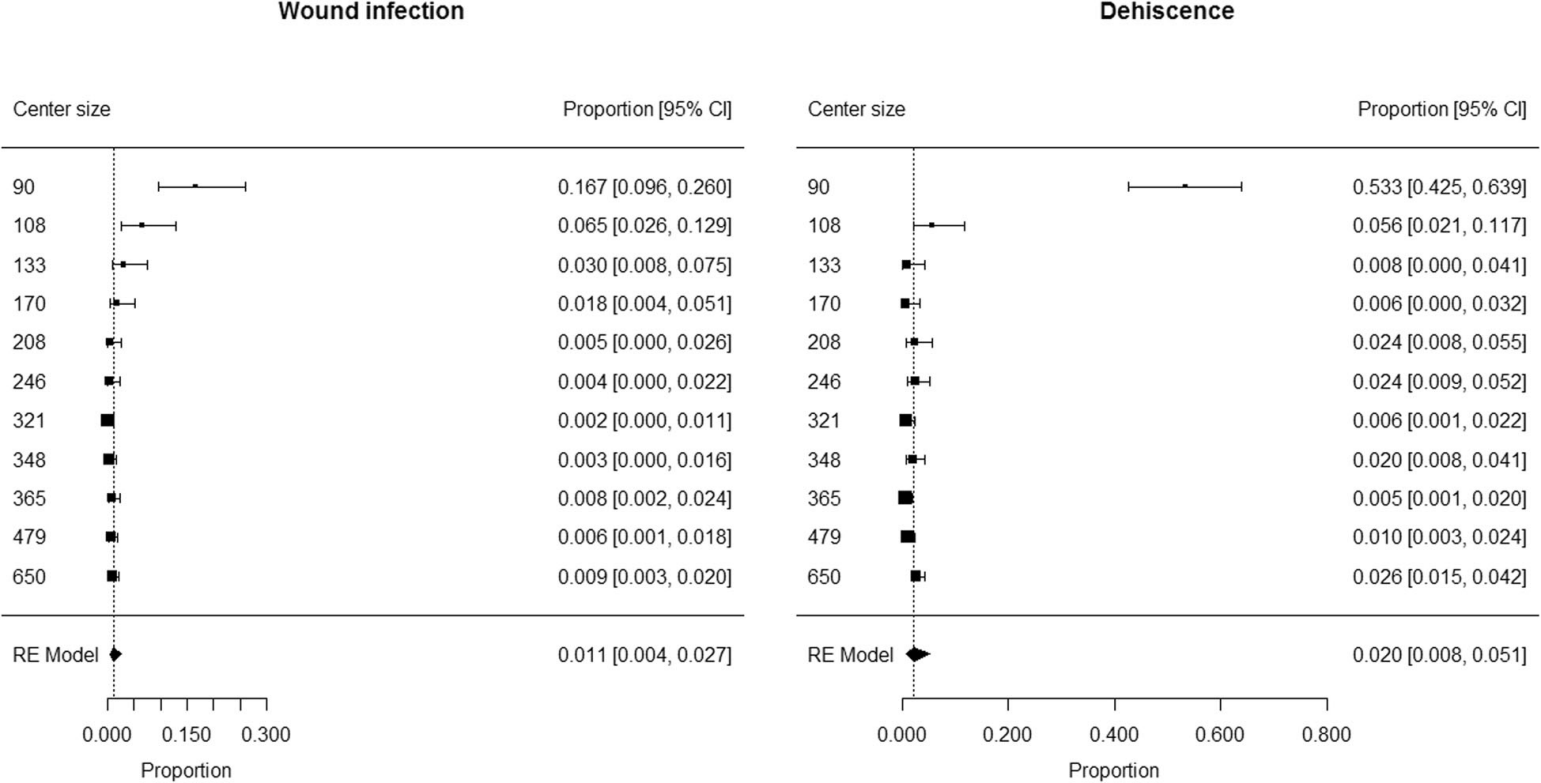
Patients developing wound infection or dehiscence after wide excision: forest plot

The proportion of patients developing wound infection ranged from 0.002 to 0.167 among centers, with a pooled proportion of 0.011 (95% CI 0.004 to 0.027) (Fig. 1). Heterogeneity was high (I^2^ = 87%) and center size (> 200 vs. < 200 patients) was found to have a significant contribution to heterogeneity (*p* < 0.0001). Summary estimates of proportion of patients developing wound infection were 0.051 (95% CI 0.021 to 0.121; I^2^ = 80%) in centers with low size (< 200 patients) and 0.006 (95% CI 0.003 to 0.009; I^2^ = 0%) in centers with large size (> 200 patients).

The proportion of patients developing dehiscence ranged from 0.005 to 0.533 among centers, with a pooled proportion of 0.020 (95% CI 0.008 to 0.051) (Fig. 1). Heterogeneity was high (I^2^ = 93%) and center size (> 100 vs. < 100 patients) was found to have a significant contribution to heterogeneity (*p* < 0.0001). Summary estimate of proportion of patients developing dehiscence was 0.016 (95% CI 0.010 to 0.024; I^2^ = 54%) in centers with large size (> 100 patients) and 0.533 (95% CI 0.425 to 0.639) in the only center with low size (< 100 patients).

### Sentinel lymph node biopsy

The analysis included 1853 patients (993 males and 860 females) who underwent SLNB in 14 centers (median 98 patients/center; min 24 patients/center, max 338 patients/center). SLNB site was axilla (1058 patients, 57.1%), neck (163 patients, 8.8%) or groin (632 patients, 34.1%). Median age was 57 years (IQR 45–69) and median BMI was 25.1 kg/m^2^ (IQR 22.7–28.0). Comorbidities were present in 304 patients (16.7%), while the information was not available in 40 patients. Overall, median Charlson Comorbidity Index was 1 (IQR 0–3).

Early wound complications occurred in 176 patients, including 29 wound infections, 39 dehiscences and 139 seromas (not mutually exclusive).

**Fig. 2:**
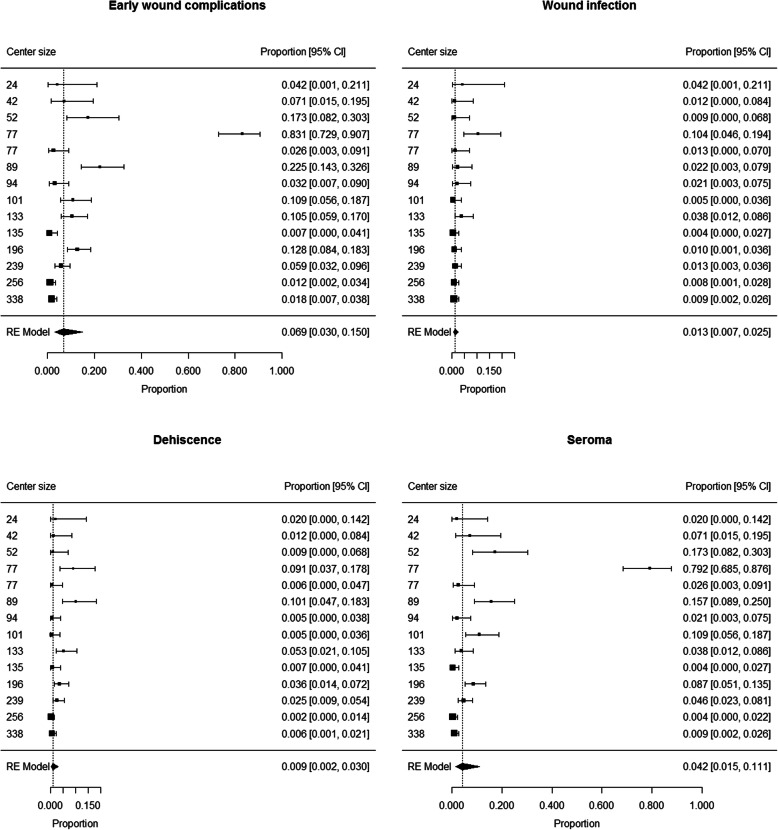
Patients developing early wound complications, wound infection, dehiscence and seroma after SLNB: forest plot

The proportion of patients developing early wound complications ranged from 0.007 to 0.831 among centers. Summary estimate of proportion of patients developing early wound complications after SLNB was 0.069 (95% CI 0.030 to 0.150) (Fig. 2). Heterogeneity was high (I^2^ = 94%); neither center size (*p* = 0.11) or rate of groin sites among SLNBs (*p* = 0.84) were not found to have a significant contribution to heterogeneity.

The proportion of patients developing wound infection ranged from 0.005 to 0.104 among centers. Summary estimate of proportion of patients developing wound infection after SLNB was 0.013 (95% CI 0.007 to 0.025) (Fig. 2). Heterogeneity was moderate (I^2^ = 60%); neither center size (*p* = 0.25) or rate of groin sites among SLNBs (*p* = 0.60) were not found to have a significant contribution to heterogeneity.

The proportion of patients developing dehiscence ranged from 0.005 to 0.101 among centers. Summary estimate of proportion of patients developing dehiscence after SLNB was 0.009 (95% CI 0.002 to 0.030) (Fig. 2). Heterogeneity was high (I^2^ = 88%); neither center size (*p* = 0.98) or rate of groin sites among SLNBs (*p* = 0.89) were not found to have a significant contribution to heterogeneity.

The proportion of patients developing seroma ranged from 0.004 to 0.792 among centers. Summary estimate of proportion of patients developing seroma after SLNB was 0.042 (95% CI 0.015 to 0.111) (Fig. 2). Heterogeneity was high (I^2^ = 95%); neither center size (*p* = 0.10) or rate of groin sites among SLNBs (*p* = 0.45) were not found to have a significant contribution to heterogeneity.

### Radical lymph node dissection

The analysis included 502 patients (301 males and 201 females) who underwent LFND in 11 centers (median 25 patients/center; min 15 patients/center, max 138 patients/center). LFND site was axilla (276 patients, 55.0%), neck (60 patients, 12.0%) or groin (166 patients, 33.0%). Median age was 59 years (IQR 47–70) and median BMI was 25.5 kg/m^2^ (IQR 22.9–28.6). Comorbidities were present in 93 patients (19.0%), while the information was not available in 12 patients. Overall, median Charlson Comorbidity Index was 2 (IQR 0–3).

Early wound complications occurred in 98 patients, including 22 wound infections, 27 dehiscences and 85 seromas (not mutually exclusive).

**Fig. 3:**
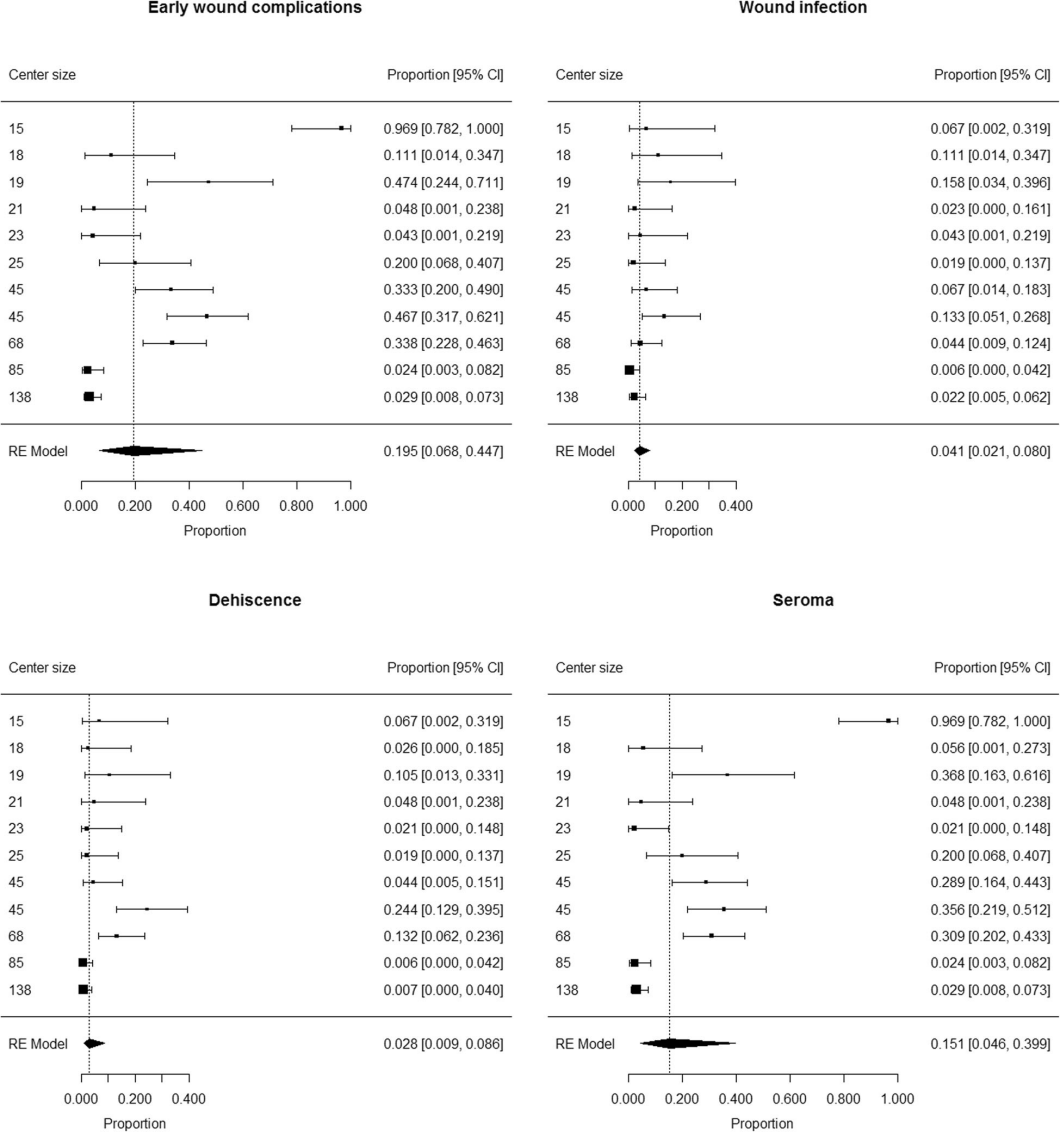
Patients developing early wound complications, wound infection, dehiscence and seroma after LFND: forest plot

The proportion of patients developing early wound complications ranged from 0.024 to 0.969 among centers. Summary estimate of proportion of patients developing early wound complications after LFND was 0.195 (95% CI 0.068 to 0.447) (Fig. 3). Heterogeneity was high (I^2^ = 94%); neither center size (*p* = 0.08) or rate of groin sites among LFNDs (*p* = 0.41) were not found to have a significant contribution to heterogeneity.

The proportion of patients developing wound infection ranged from 0.006 to 0.58 among centers. Summary estimate of proportion of patients developing wound infection after SLNB was 0.041 (95% CI 0.021 to 0.080) (Fig. 3). Heterogeneity was moderate (I^2^ = 54%); neither center size (*p* = 0.07) or rate of groin sites among SLNBs (*p* = 0.53) were not found to have a significant contribution to heterogeneity.

The proportion of patients developing dehiscence ranged from 0.006 to 0.244 among centers. Summary estimate of proportion of patients developing dehiscence after SLNB was 0.028 (95% CI 0.009 to 0.086) (Fig. 3). Heterogeneity was high (I^2^ = 80%); neither center size (*p* = 0.38) or rate of groin sites among SLNBs (*p* = 0.14) were not found to have a significant contribution to heterogeneity.

The proportion of patients developing seroma ranged from 0.021 to 0.969 among centers. Summary estimate of proportion of patients developing seroma after SLNB was 0.151 (95% CI 0.046 to 0.399) (Fig. 3). Heterogeneity was high (I^2^ = 95%); neither center size (*p* = 0.18) or rate of groin sites among SLNBs (*p* = 0.29) were not found to have a significant contribution to heterogeneity.

## Discussion

This study reported the morbidity rates in the largest series of non-metastatic melanoma patients in Italy and one of the largest series worldwide. The present work allowed to evaluate standardization and quality of surgical treatment of cutaneous melanoma within the frame of the Italian Melanoma Intergroup, which is the largest Italian scientific organization dedicated to the management of patients with this disease [10].

The occurrence of wound dehiscence and seroma after SLNB or LFND were in broad agreement with previous studies [13–15] (Table 1), thus suggesting limited opportunities for further improvements now. Such rates can be used as morbidity benchmarks in addition to available quality indicators for surgical treatment of melanoma [10].

**Table 1:** Referral values for morbidity rate in the IMI-CNMR study and in the international

Surgical	Indicators	Benchmark referral values	
Procedure		Present study	International literature
**WE**	Wound infection	1.1% (0.4 to 2.7%)	4.6–8.4%^a^
	Wound dehiscence	2.0% (0.8 to 5.1%)	3.5–4.6%^a^
	Skin graft failure	unreliable	< 2%^a^
**SLNB**	Wound Infection	1.3% (0.7 to 2.5%)	2.9% (1.5 to 4.6%)^b^
	Wound dehiscence	0.9% (0.2 to 3.0%)	0.24–1.2%^a^
	Seroma	4.2% (1.5 to 11.1%)	5.1% (2.5 to 8.6%)^b^
**LFND**	Wound infection	4.1% (2.1 to 8.0%)	15.8%^a^
	Wound dehiscence	2.8% (0.9 to 8.6%)	3%^a^
	Wound infection and/or dehiscence	6.5% (2.9 to 14.0%)	21.6% (13.8 to 30.6%)^c^
	Seroma	15.1% (4.6 to 39.9%)	17.9% (10.3 to 27%)^c^

Data expressed as percentage with 95% confidence interval in parentheses

^a^Morton DL, Cochran AJ, Thompson JF, Elashoff R, Essner R, Glass EC, Mozzillo N, Nieweg OE, Roses DF, Hoekstra HJ, Karakousis CP, Reintgen DS, Coventry BJ, Wang HJ; Multicenter Selective Lymphadenectomy Trial Group. Sentinel node biopsy for early-stage melanoma: accuracy and morbidity in MSLT-I, an international multicenter trial. Ann Surg. 2005 Sep;242 (3):302–11; discussion 311–3

^b^Moody JA, Ali RF, Carbone AC, Singh S, Hardwicke JT. Complications of sentinel lymph node biopsy for melanoma-A systematic review of the literature. Eur J Surg Oncol. 2017;43:270–277

^c^Moody JA, Botham SJ, Dahill KE, Wallace DL, Hardwicke JT. Complications following completion lymphadenectomy versus therapeutic lymphadenectomy for melanoma - A systematic review of the literature. Eur J Surg Oncol. 2017;43:1760–1767

Interestingly, we found lower occurrence of wound infection after WE or LFND when compared to available literature [13] (Table 1). On one hand, this difference might be partially due to different definitions of wound infection, i.e. presence of fever, only skin redness, suppuration versus cellulitis, isolation of bacteria from wound. On the other hand, some factors might have led to such difference, including: i) recording of infections occurring only during hospital stay or also at the time of outpatient department visits; ii) different frequency/type of antibiotic prophylaxis; iii) different surgical techniques (i.e., use, type and duration of permanence of drainages in the surgical wound).

Postoperative infections account for around one out of four complications associated with hospital-related health care procedures, and can impair patient prognosis [16]. Beyond clinical importance, a low infection rate can also have an economic impact, since the management of hospital-related infections requires about 0.8% of gross domestic product (GDP) in Italy [17].

Unfortunately, the information on skin graft failure was not reliable because one out of five forms did not report such complication. This situation likely occurred because skin graft failure required a reconstructive surgical management that several centers demanded to a different surgical unit. This situation may be addressed by improving the information exchange among surgical centers involved in patient care.

Of note, morbidity rates showed high heterogeneity across melanoma centers, underlying the role of the center itself on this matter. Our data suggested an association between higher morbidity rate and small-volume centers, thus confirming the relationship between patient outcome and hospital surgical volume [18].

The present study contributes to the definition of quality indicators for surgical treatment for non-metastatic skin melanoma, by adding morbidity indicators that can be used as the basis for a standardized quality assurance program [10]. The importance of this topic relies on the large number of surgical procedures for non-metastatic skin melanoma [2], thus patient management and prognosis can benefit from quality control and standardization of such procedures [4, 5].

The strengths of this study included the prospective collection of data in a national registry, the multicenter design and the standardized definitions of the complications [11]. The study has some limitations. First, a considerable number of centers were excluded due to poor completeness of data. Although this approach allowed limiting the impact of low-quality data on the study results, the representativeness of the included centers may be limited. Future developments will aim to achieve adequate completeness of data in the excluded centers and will implement regular audits. Second, the occurrence on skin graft failure after WE could not be evaluated due to the non-negligible number of unfilled forms. This limitation can be addressed by future improvements regarding information exchange among surgical centers involved in patient care.

## Conclusion

Our findings contribute to available literature in setting up the recommended standards for melanoma centers, thus improving the quality of surgery offered to patients. Such quality indicators can be used by other hospitals to direct quality improvement efforts.

## Supplementary Information

**Additional file 1: Supplementary Figure 1.** Flow-chart of patient inclusion in wide excision (WE) analysis. **Supplementary** **Figure 2.** Flow-chart of patient inclusion in sentinel lymph node biopsy (SLNB) analysis. **Supplementary Figure 3.** Flow-chart of patient inclusion in radical lymph node dissection (LFND) analysis.

## Data Availability

The datasets generated and analysed during the current study are available on Mendeley Data. Del Fiore, Paolo (2020), “Morbidity melanoma data”, Mendeley Data, V2, doi: 10.17632/vt2n4kwb25.210.17632/vt2n4kwb25.2

## Abbreviations

WE: Wide excision
SLNB: Sentinel lymph node biopsy
LFND: Radical lymph node dissection
IMI: Italian Melanoma Intergroup
CNMR: Clinical National Melanoma Registry
GDP: Gross domestic product
