# Supplementary material for: The surgical treatment of non-metastatic melanoma in a Clinical National Melanoma Registry Study Group (CNMR): a retrospective cohort quality improvement study to reduce the morbidity rates
Source: BMC Cancer. 2021 Jan 5;21:8. doi: 10.1186/s12885-020-07705-4 (PMC7786513; doi:10.1186/s12885-020-07705-4)
Supplement: Supplementary file 1 — Additional file 1: Supplementary Figure 1. Flow-chart of patient inclusion in wide excision (WE) analysis. Supplementary Figure 2. Flow-chart of patient inclusion in sentinel lymph node biopsy (SLNB) analysis. Supplementary Figure 3. Flow-chart of patient inclusion in radical lymph node dissection (LFND) analysis. [file 12885_2020_7705_MOESM1_ESM.docx]

**Supplementary** **Figure 1**. Flow-chart of patient inclusion in wide excision (WE) analysis


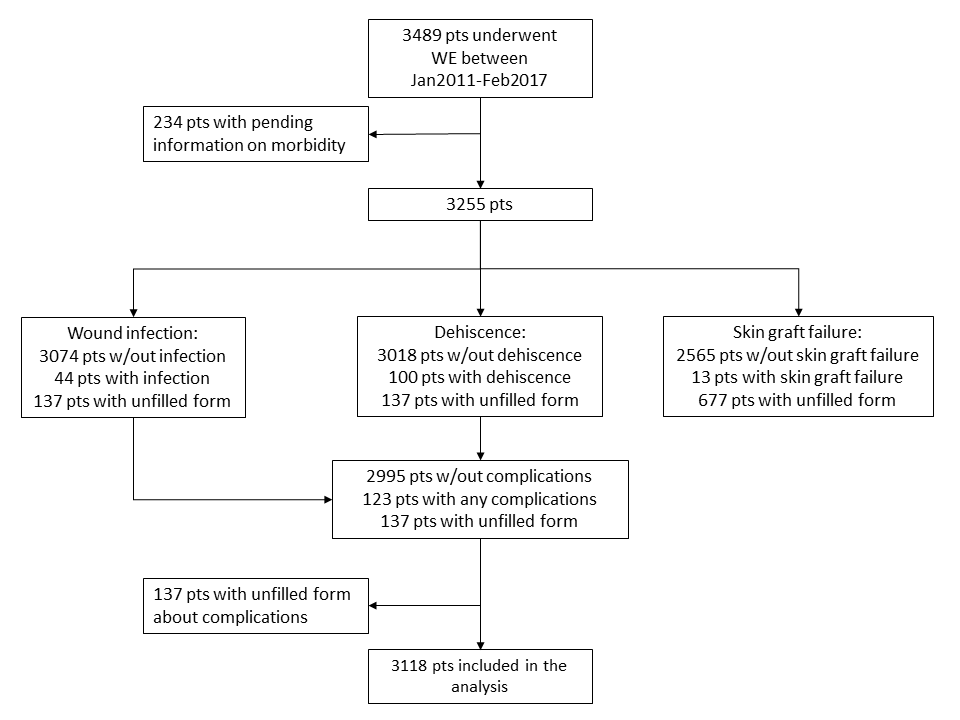


**Supplementary** **Figure 2**. Flow-chart of patient inclusion in sentinel lymph node biopsy (SLNB) analysis


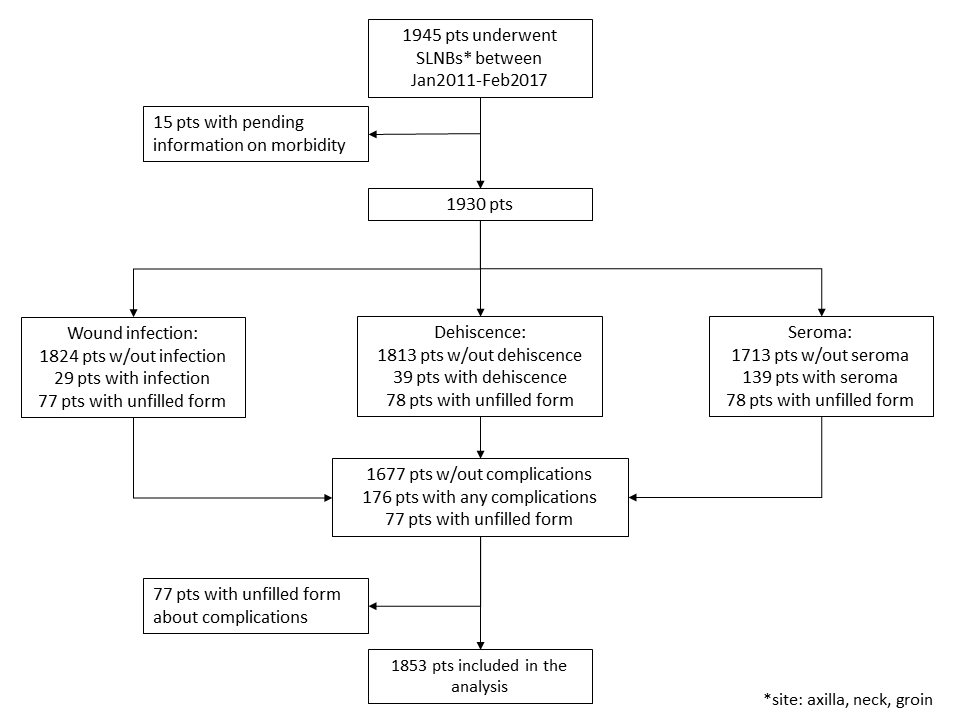


**Supplementary** **Figure 3**. Flow-chart of patient inclusion in radical lymph node dissection (LFND) analysis

**
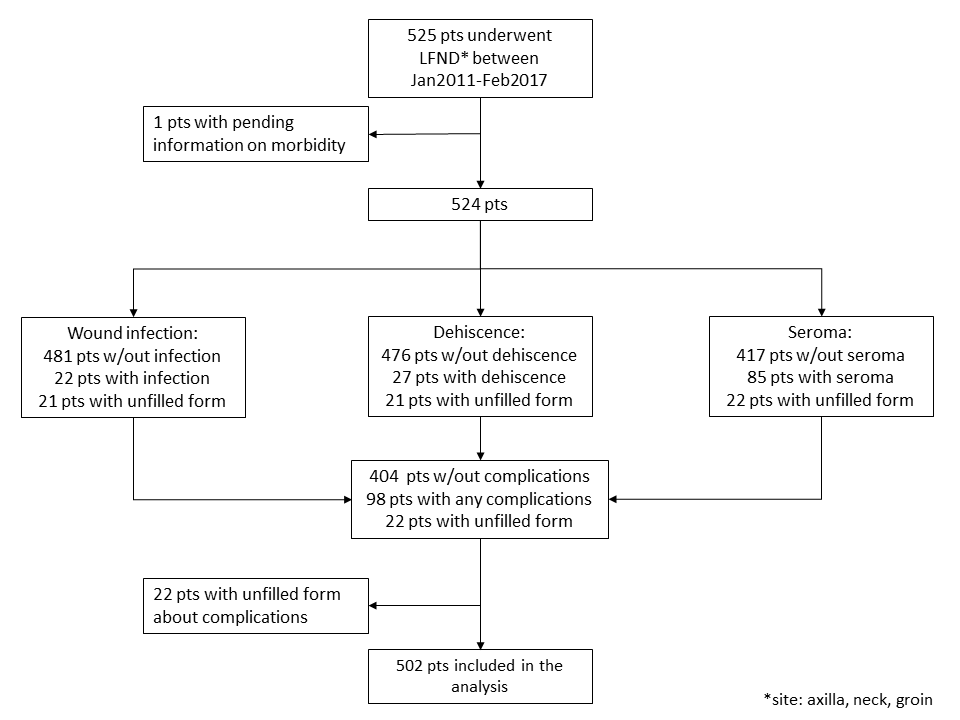
**
